# Complete Genome Sequence of *Mycobacterium* *chimaera* Strain CDC2015-22-71

**DOI:** 10.1128/genomeA.00693-17

**Published:** 2017-08-03

**Authors:** Nabeeh A. Hasan, Adrian Lawsin, K. Allison Perry, Efe Alyanak, Nadege C. Toney, Allyson Malecha, Lori A. Rowe, Dhwani Batra, Heather Moulton-Meissner, Jeffrey R. Miller, Michael Strong, Alison Laufer Halpin

**Affiliations:** a Center for Genes, Environment and Health, National Jewish Health, Denver, Colorado, USA; b Division of Healthcare Quality Promotion, National Center for Emerging and Zoonotic Infectious Diseases, CDC, Atlanta, Georgia, USA; c Office of Public Health Preparedness and Response, Division of State and Local Readiness, CDC, Atlanta, Georgia, USA; d Pennsylvania Department of Health, Harrisburg, Pennsylvania, USA

## Abstract

*Mycobacterium chimaera* is a nontuberculous mycobacterium species commonly found in the environment. Here, we report the first complete genome sequence of a strain from the investigation of invasive infections following open-heart surgeries that used contaminated LivaNova Sorin Stockert 3T heater-cooler devices.

## GENOME ANNOUNCEMENT

*Mycobacterium chimaera* is a nontuberculous mycobacterium (NTM) species within the *Mycobacterium avium* complex (MAC). *M. chimaera* is an emerging cause of infection, in particular due to increasing awareness of infections linked with exposure to contaminated LivaNova Sorin Stockert 3T heater-cooler unit (HCU) devices (1–12). Despite the public health relevance of *M. chimaera*, no publicly available genome is representative of genotypes implicated in HCU contamination (13, 14). We present this here, using CDC2015-22-71, a clinical isolate from a Pennsylvania patient epidemiologically linked to a cluster of *M. chimaera* infections following exposure to an HCU.

Genomic DNA was extracted from CDC2015-22-71 grown in 7H9 broth (BD, Franklin Lakes, NJ, USA) at 36°C for approximately 1 week. The genome was sequenced using the Pacific Biosciences RSII (PacBio, Menlo Park, CA, USA) and Illumina MiSeq (San Diego, CA, USA) platforms. A 10-kb library was generated with the SMRTbell template prep kit 1. The library was then bound to polymerase using the DNA/polymerase binding kit P6v2. The bound library was loaded on two SMRTcells and sequenced with C4v2 chemistry for 360-min movies on the RSII instrument. Sequence reads were filtered and assembled *de novo* utilizing the PacBio HGAP version 3 (15). The PacBio assembly was corrected with Illumina reads using Pilon version 1.20 (16). The resulting assembly was compared to other *M. chimaera* genomes using Mauve version 2.3.1 (17). Genomic features were identified and annotated using the NCBI Prokaryotic Genome Annotation Pipeline.

The genome of *M. chimaera* CDC2015-22-71 consists of four scaffolds, equaling 6,247,640 bp (6,078,351-bp chromosome; 97,267-bp plasmid; 39,887-bp plasmid; 32,135-bp plasmid) and a G+C content of 67.6%. A total of 5,795 coding sequences were predicted, including 5,627 protein-coding genes and 168 pseudogenes. Our genome assembly contains 47 tRNAs, 3 noncoding RNAs, and 1 rRNA cistron consisting of the 16S, 23S, and 5S rRNA genes.

Whole-genome alignments of *M. chimaera* CDC2015-22-71, Hawaiian respiratory strain AH16 (GenBank accession number CP012885 [13]), and Irish respiratory strains MCIMRL6, MCIMRL4, and MCIMRL2 (GenBank accession numbers LJHN00000000, LJHM00000000, and LJHL00000000 [14]) revealed 15,077 single-nucleotide polymorphisms (SNPs) compared to AH16; 8,870 SNPs compared to MCIMRL2; 2,229 SNPs compared to MCIMRL4; and 8,098 SNPs compared to MCIMRL6. Comparison of gene content between the five *M. chimaera* genomes revealed a core gene set of 4,726 genes (83.9% of the 2015-22-71 genome [18]). The comparison of CDC2015-22-71 against all previously identified *M. chimaera* genomes resulted in average nucleotide identity values greater than or equal to 98.70%, which are greater than the 95 to 96% cutoff for species boundaries (19).

This *M. chimaera* genome assembly is the first complete NTM genome associated with an outbreak and will serve as a reference for epidemiological investigations of North American-based HCU contamination and postsurgical *M. chimaera* infections.

### Accession number(s).

The genome sequence of *M. chimaera* CDC2015-22-71 has been deposited in NCBI GenBank under accession numbers CP019221 through CP019224. PacBio and Illumina reads have been deposited in NCBI under BioProject number PRJNA344472 and BioSample number SAMN05824346.
